# A comparative study of movement characteristics and neuromuscular responses in tennis one-handed backhand strokes under three self-resistance stroke types

**DOI:** 10.3389/fphys.2026.1921253

**Published:** 2026-09-15

**Authors:** Ziwen Wang, Kaidi Zhang, Zichen Jin, Jie Ren, Weiguo Liu

**Affiliations:** 1 College of Physical Education and Health, Guangxi Normal University, Guilin, China; 2 Department of Physical Education, Guilin Medical University, Guilin, China

**Keywords:** One-handed backhand, self-resistance stroke types, neuromuscular activation, surface electromyography, comparative analysis

## Abstract

**Objective:**

The aim of this study was to investigate how different self-resistance strokes influence neuromuscular activation patterns and immediate stroke performance during the one-handed backhand stroke.

**Methods:**

Using 3D image analysis system and Noraxon surface electromyography (EMG) system, we quantified movement rhythm, structure, and effectiveness of the three different one-handed backhand strokes performed by 30 male tennis players, as well as the activation sequence and integrated EMG parameters of eight prime movers. We employed one-way analysis of variance (ANOVA) and multiple comparisons to examine differences in the movement characteristics among the three stroke types.

**Results:**

1) No statistically significant differences were detected in the structural kinematic variables among the three strokes. But some differences were observed in the rhythm of the movements, with rotational phase of the stroke being the fastest in the brief self-resistance stroke; 2) In terms of performance outcomes, brief self-resistance stroke exhibited the highest racket head speed and ball speed, prolonged self-resistance stroke had high racket head speed but low ball speed, and free self-resistance stroke had low racket head speed and low ball speed; 3) The sequence of muscle activation was divided into two groups for the free self-resistance stroke and three groups for the stroke. There were no differences among the three strokes in the integrated electromyographic (EMG) values of the prime movers responsible for ball strokes and body rotation; the integrated EMG values of the prime movers involved in physical stroke were higher in the two self-resistance strokes than in the free self-resistance stroke; and the integrated EMG values of the quadriceps muscles were highest in the brief self-resistance stroke.

**Conclusion:**

1) Movement patterns were similar among strokes, while brief self-resistance stroke showed faster body rotation and improved stroke performance. 2) Brief self-resistance stroke achieved faster rotational execution and superior stroke performance, which may be associated with greater cumulative neuromuscular activity. Further studies using time-normalized EMG and kinetic measurements are needed to clarify the underlying mechanisms. 3) Brief self-resistance strokes showed faster rotational execution and higher acute stroke performance, which may be associated with altered neuromuscular activation patterns.

## 1 Introduction

The backhand stroke is one of the three fundamental techniques in tennis (Genevois et al., 2015) and plays a crucial role in determining a player’s ability to transition between offense and defense, as well as the outcome of a match. Given the trend toward higher speed and greater power in modern competitive tennis, players must complete rapid movement, body adjustment, and precise shots within a limited reaction time, placing higher demands on the stability and aggressiveness of their backhand technique (Chan et al., 2022). Furthermore, since most players rely on their forehand as their primary means of scoring, the backhand side often becomes a key target for opponents during matches; consequently, the level of backhand skill has become an important indicator of a player’s overall competitive ability (Genevois et al., 2015). Within backhand techniques, the one-handed backhand and the two-handed backhand are the two primary forms (Reid and Elliott, 2002). Compared to the two-handed backhand, the one-handed backhand offers greater unpredictability and versatility (Knudson and Blackwell, 1997). Currently, some top players in men’s professional tennis still use the one-handed backhand, which stands out for its creativity in attack and its visual appeal (Akutagawa and Kojima, 2005). Since the one-handed backhand relies solely on one upper limb to control the racket face and exhibit elevated neuromuscular activation, it places higher demands on the player’s neuromuscular coordination and upper-body strength (Stępień et al., 2011). Research has shown that muscle activation patterns play a particularly important role in technical performance (Giangarra et al., 1993). Different stroke patterns often correspond to distinct muscle activation patterns, and differences in these patterns may affect stroke speed, stability, and overall performance (Tai et al., 2022). Currently, research on the one-handed backhand in tennis, both domestically and internationally, has largely focused on the optimization of kinematic parameters, biomechanical analysis of injuries, or comparisons between one- and two-handed backhand techniques (Knudson and Blackwell, 1997; Glynn, 2007; Genevois et al., 2015; Kachel et al., 2015; Reid et al., 2015; Ruiz-Malagón et al., 2022). Few studies have systematically compared the differences in neuromuscular activation characteristics and kinematic performance among various stroke patterns from the perspective of self-resistance strokes.

Based on this, this study designed three representative tennis one-handed backhand strokes: the free self-resistance stroke (where the non-racket hand lightly rests on the racket handle before releasing it, allowing the racket hand to strike naturally); the prolonged self-resistance stroke (where the non-racket hand and racket hand exert continuous, stable opposing force, and after release, the racket hand strokes to strike the ball); and brief self-resistance stroke (where the non-dominant hand exerts a brief, forceful counterforce against the dominant hand, and upon rapid release, the dominant hand strokes to strike the ball). From a physiological perspective, self-resistance applied before stroke execution may modify the preparatory state of the neuromuscular system. These three self -resistance strokes were developed as experimental movement strategies to examine how different resistance characteristics may influence acute neuromuscular control and stroke performance.

## 2 Materials and methods

### 2.1 Participants

This study used G*Power 3.1 software to estimate the required sample size for a within-subject repeated-measures ANOVA design. The calculation was based on a medium effect size (f = 0.25), an alpha level of 0.05, and statistical power of 0.80. Because no single primary outcome was prespecified before data collection, the sample-size estimation was based on the general repeated-measures design rather than a specific outcome variable. The analysis assumed a correlation of 0.5 among repeated measurements and a nonsphericity correction factor (ϵ) of 0.8. The calculation indicated that at least 28 participants were required. This study included 30 male tennis players as participants. All participants had regular tennis training experience and were familiar with the one-handed backhand technique. All participants were right-handed, with their backhand hitting zone located on the left side of their torso, and all employed a semi-open step-in stance for their one-handed backhand technique. None of the participants reported upper or lower limb injuries within the previous six months. The study complied with the Declaration of Helsinki and was approved by the Ethics Committee of the Guangxi Normal University of China (approval No. 20250511002). All study participants signed an informed consent form before participation. Basic information is shown in **Table 1**.

**Table 1:** Basic information on athletes.

Age (years)	Height (cm)	Body mass(kg)	Training experience (years)
22 ± 3	175 ± 10	65 ± 7	5 ± 3

### 2.2 Testing equipment

Noraxon Surface Electromyography (EMG) Testing System (manufactured in the U.S.), two JVC high-speed cameras (200 frames per second), a laptop, disposable electrode pads, elastic bandages, alcohol swabs, razors, etc.

### 2.3 Testing methods

Kinematics Testing: Two JVC high-speed cameras (200 Hz) were positioned diagonally in front of and behind the participant’s left side to capture the one-handed backhand stroke from two different perspectives. Prior to data collection, a calibration frame with known dimensions was placed within the hitting area to establish the global coordinate system and enable three-dimensional reconstruction. The two camera views were synchronized using the racket–ball contact event as a common temporal reference point. Ball contact was identified manually by the investigator based on the first frame in which the racket head visibly struck the ball. The stroke frame was independently reviewed twice by the same investigator, and ambiguous trials were rechecked using slow-motion playback to minimize event-identification error. Anatomical landmarks were manually identified and digitised frame-by-frame using SIMI Motion software (SIMI Reality Motion Systems GmbH, Germany). The digitised coordinates were reconstructed into three-dimensional kinematic coordinates using the calibrated multi-camera reconstruction function of SIMI Motion software. The coordinate system was defined as follows: the X-axis represented the mediolateral direction, the Y-axis represented the anteroposterior direction, and the Z-axis represented the vertical direction.

EMG Testing: Surface EMG signals were recorded using the Noraxon EMG system (Noraxon USA Inc., USA) at a sampling frequency of 2000 Hz. Eight muscles on the racket-holding side were selected based on their functional involvement during the one-handed backhand stroke: posterior deltoid, brachioradialis, triceps brachii, upper trapezius, erector spinae, quadriceps femoris of the supporting leg, quadriceps femoris of the pushing leg, and contralateral external oblique. Before the stroke trials, participants performed maximal voluntary contraction (MVC) tests for each selected muscle to obtain reference EMG values for normalization. MVC procedures were performed according to standardized muscle-specific positions, with participants instructed to gradually show elevated neuromuscular activation to maximal effort and maintain the contraction for approximately 3–5 s. Verbal encouragement was provided during each trial. The highest RMS EMG value obtained during MVC trials was used as the reference value for normalization. Bipolar Ag/AgCl electrodes were placed according to the SENIAM recommendations. Before electrode placement, the skin was shaved, lightly abraded, and cleaned with alcohol to reduce impedance. Electrodes were positioned parallel to the muscle fiber direction with an inter-electrode distance of 20 mm. A reference electrode was placed over an electrically neutral bony area. To minimize cross-talk, electrode placement was carefully checked according to anatomical landmarks and muscle contraction tests were performed before data collection. The EMG recordings were synchronized with the video recordings using the racket–ball contact event as a common temporal reference point. The contact frame was identified from the synchronized video recordings and was used to align the timing of movement phases and EMG activation events. Because synchronization was based on manual event identification rather than hardware triggering, potential temporal uncertainty related to frame resolution and event detection was acknowledged.

Experimental Procedure: To minimize errors, all balls were delivered uniformly by a ball machine with consistent delivery strokes throughout the experiment. Participants used their own competition rackets during testing to ensure familiarity with the equipment. The same racket was used by each participant across all experimental strokes. For each movement test, athletes were required to start from the center point and move into the hitting zone using their usual footwork. They then returned the ball to the designated landing area using three different closed-stance one-handed backhand techniques. To minimize potential order and learning effects, the sequence of the three experimental strokes (Free self-resistance stroke, Prolonged self-resistance stroke, and brief self-resistance stroke) was randomized for each participant using a computer-generated random number sequence before the start of the testing session. Prior to formal testing, all participants completed a familiarization session consisting of multiple practice strokes per condition. None of the participants had previous systematic training experience with these self-resistance stroke strokes. Therefore, the familiarization session was conducted to ensure that participants understood the experimental instructions and could consistently reproduce the required movement strategies before data collection. During the formal test, each participant performed a fixed number of 10 valid strokes per stroke. A 10-minute rest interval was given between different strokes to allow for full neuromuscular recovery and to minimize fatigue carryover effects. Within each condition, participants were instructed to perform each stroke with their maximal intended stroke effort while maintaining technical accuracy and consistency. Approximately 20–30 seconds were provided between attempts. All recorded strokes were independently evaluated by two certified tennis coaches who were blinded to the experimental strokes. Only those strokes that were rated as technically sound by both coaches were retained for further analysis. In cases of disagreement, a third senior expert was consulted to make the final decision. Any stroke that missed the designated target area, resulted in a loss of balance, or caused subjective discomfort was discarded and repeated. In this study, three types of tennis one-handed backhand strokes were designed based on different self-resistance strategies involving the non-racket-holding hand: The free self-resistance stroke required participants to lightly place the non-racket-holding hand on the racket handle before releasing it, allowing the racket-holding hand to complete the stroke without intentional resistance from the non-racket-holding hand; The prolonged self-resistance stroke required participants to grip the racket handle with the non-racket-holding hand and apply prolonged self-resistance against the racket-holding hand before release. After maintaining this perceived sustained resistance, participants released the racket and completed the one-handed backhand stroke; the brief self-resistance stroke required participants to apply a brief self-resistance with the non-racket-holding hand against the racket-holding hand before quickly releasing the racket and completing the stroke. Prolonged self-resistance was defined as maintaining resistance for > 2 seconds before release; brief self-resistance was defined as maintaining resistance for< 0.5 second.

### 2.4 Definition of the motion phase

This study divides the overall one-handed backhand technique in tennis into three phases: the step-in and racket-back phase, the body rotation and contact phase, and the follow-through phase. The step-in and racket-back phase begins with the ready position; the player steps forward with the right foot, pointing the toes toward the incoming ball while rotating the shoulders and bringing the racket back, until both the player’s shoulders and the bottom of the racket handle are facing the net directly ahead; The body rotation and stroke phase refers to the period after the racket draw is completed, during which the racket begins to accelerate toward the incoming ball, accompanied by the athlete’s hip and shoulder rotation, which drives the racket’s rotation, until the moment the racket head makes contact with the ball; the follow-through phase refers to the period from the moment the racket head makes contact with the ball until the athlete strokes the racket to its highest point. All movement phases were defined according to observable mechanical events identified from synchronized video recordings. The beginning and ending frames of each phase were determined as follows: the step-in and racket-back phase started from the ready position and ended when the racket preparation was completed; the body rotation and contact phase started immediately after racket preparation and ended at the first racket–ball contact frame; the follow-through phase started at racket–ball contact and ended when the racket reached its highest point after impact.

### 2.5 Data processing and statistical analysis

SIMI Motion software (SIMI Reality Motion Systems GmbH, Germany) was used for three-dimensional reconstruction and frame-by-frame digitization of the recorded images. The reconstructed three-dimensional coordinates of anatomical landmarks were used to obtain kinematic parameters, including movement rhythm, contact position, body posture, and stroke performance. The digitized landmarks included the head, shoulder, elbow, wrist, hip, knee, ankle, toe, racket head, and ball position. Movement rhythm was determined based on the duration of each predefined stroke phase. The contact position was calculated from the three-dimensional coordinates of the racket–ball contact point relative to the supporting foot and normalized by participant height. Body posture was evaluated using the estimated relative height of the center of gravity, knee angles of the supporting and pushing legs, elbow angle of the racket-holding arm, and shoulder angle of the racket-holding arm. Joint angles were calculated from the corresponding anatomical landmark vectors. The relative height of the center of gravity was obtained from the reconstructed body coordinates and normalized to participant height. Stroke performance was assessed by ball speed and racket head speed; Ball speed was calculated from the displacement of the ball during the initial post-impact flight period using consecutive digitized frames immediately after racket–ball contact. Racket head speed was calculated from the displacement of the racket head over consecutive frames immediately before impact. Velocity was derived from displacement divided by the corresponding time interval determined by the camera sampling frequency (200 Hz). No additional smoothing filter was applied to the kinematic data because the high-speed camera sampling frequency provided sufficient temporal resolution for identifying the rapid stroke events. All video sequences were digitized by the same investigator using a standardized procedure to minimize measurement variability. For each participant and each condition, all kinematic and electromyographic parameters were averaged across all retained valid trials to obtain a single representative value for subsequent statistical analysis. The raw EMG signals were processed using Noraxon software. Signals were band-pass filtered (20–450 Hz), full-wave rectified, and smoothed using a root-mean-square algorithm with a 50-ms window. The processed EMG amplitudes were normalized to the maximum RMS EMG value obtained during MVC trials and expressed as a percentage of MVC (%MVC). The onset of muscle activation was identified using a threshold-based method. The baseline EMG activity was determined during the 200 ms period immediately preceding the initiation of the rotational stroke phase. Muscle activation onset was defined as the first point at which the rectified EMG signal exceeded three standard deviations above the baseline mean and remained above this threshold for at least 25 ms. The onset timing of each muscle was calculated relative to the beginning of the rotational stroke phase identified from synchronized video recordings. When two muscles showed activation onset times within 10 ms of each other, they were considered to have simultaneous activation and were assigned to the same activation cluster. Integrated EMG (iEMG) was calculated by integrating the normalized EMG signal across the entire duration of each movement phase. Because the duration of each phase differed among strokes, iEMG values were interpreted as representing the overall neuromuscular demand during each phase rather than muscle activation amplitude alone. Data analysis was performed using SPSS 16.0 statistical software, and all data are presented as mean ± standard deviation (Mean ± SD). A one-way repeated-measures analysis of variance (ANOVA) was primarily used to statistically compare differences in the relevant technical parameters of the three stroke techniques. For muscle activation sequence analysis, activation onset time was first treated as a continuous variable. Pairwise comparisons were performed based on activation time values, and muscles showing no statistically significant differences in activation onset time were grouped into the same activation cluster. The activation sequence was subsequently derived according to the temporal order of these clusters rather than by treating sequence ranks as continuous variables. Prior to repeated-measures ANOVA, the normality of data distribution was assessed using the Shapiro–Wilk test. The sphericity assumption was evaluated with Mauchly’s test, and the results indicated that the assumption was met for all dependent variables (all p > 0.05); therefore, no corrections to the degrees of freedom were required. For the three strokes within-subject comparisons, the repeated-measures ANOVA was conducted with *d_effect_* = 2 and *d_error_* = 58. For significant main effects, pairwise comparisons were conducted using the Holm–Bonferroni correction to control for multiple comparisons. Effect sizes were reported as partial eta squared ( $\eta_{p}^{2}$). The significance level was set at *α* = 0.05.

## 3 Results

### 3.1 A comparative kinematic analysis of three self-resistance strokes

#### 3.1.1 Comparison of movement rhythms

The repeated-measures ANOVA showed that no statistically significant difference was observed in the duration of the footwork and backswing phase among the three strokes (*F* = 2.13, *P* = 0.09, *ηp*^2^ = 0.07). No statistically significant differences were observed in total stroke duration among the three strokes (*F* = 3.01, *P* = 0.07, ηp^2^ = 0.09). Statistically significant main effects were found for the rotational phase (*F* = 13.41, *P* < 0.001, *ηp*^2^ = 0.49) and follow-through phase *(F* = 4.88, *P* = 0.03, *ηp*^2^ = 0.25). Holm–Bonferroni-adjusted pairwise comparisons indicated that the duration of the rotational phase was shorter in the brief self-resistance stroke than in both the free self-resistance stroke (mean difference = -0.12, 95% CI: -0.15 ~ -0.09, *P* < 0.05) and prolonged self-resistance stroke (mean difference = -0.06, 95% CI: -95% CI:-0.09 ~ -0.03, *P* < 0.05). In addition, the follow-through phase duration was longer in the free self-resistance stroke than in the brief self-resistance stroke (mean difference = 0.15, 95% CI:0.05 ~ 0.25, *P* < 0.05). See **Figure 1**.

**Figure 1:**
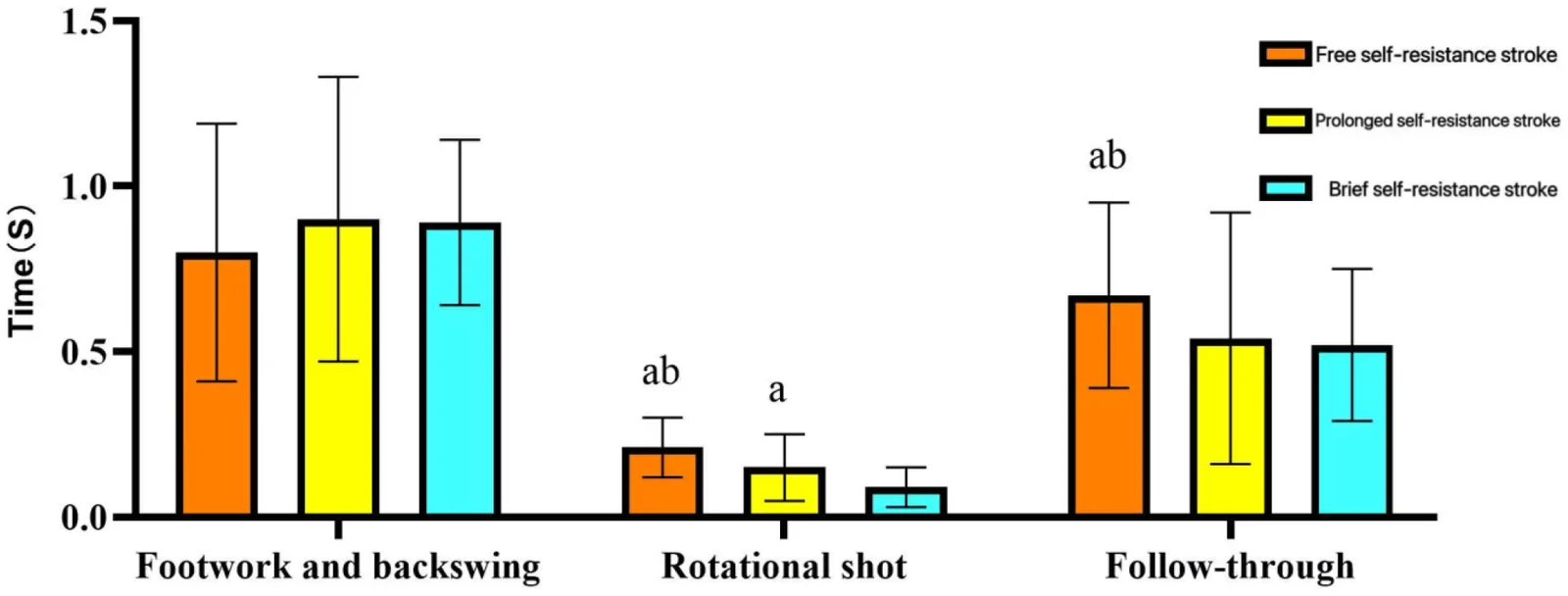
Comparison of time distribution for three stroke types. Note: "a" indicates a significant difference compared to brief self-resistance stroke; "b" indicates a significant difference compared to prolonged stroke; "c" indicates a significant difference compared to free self-resistance stroke; Error bars represent standard deviations (SD). The same applies below.

#### 3.1.2 Comparison of point of contact

No statistically significant differences were observed in the racket-ball contact position among the three strokes in the mediolateral direction (*F* = 1.89, *P* = 0.23, *ηp*^2^ = 0.06), anteroposterior direction (*F* = 1.77, *P* = 0.43, *ηp*^2^ = 0.06), or vertical direction (*F=*3.16, *P* = 0.07, *ηp*^2^ = 0.10). See **Figure 2**.

**Figure 2:**
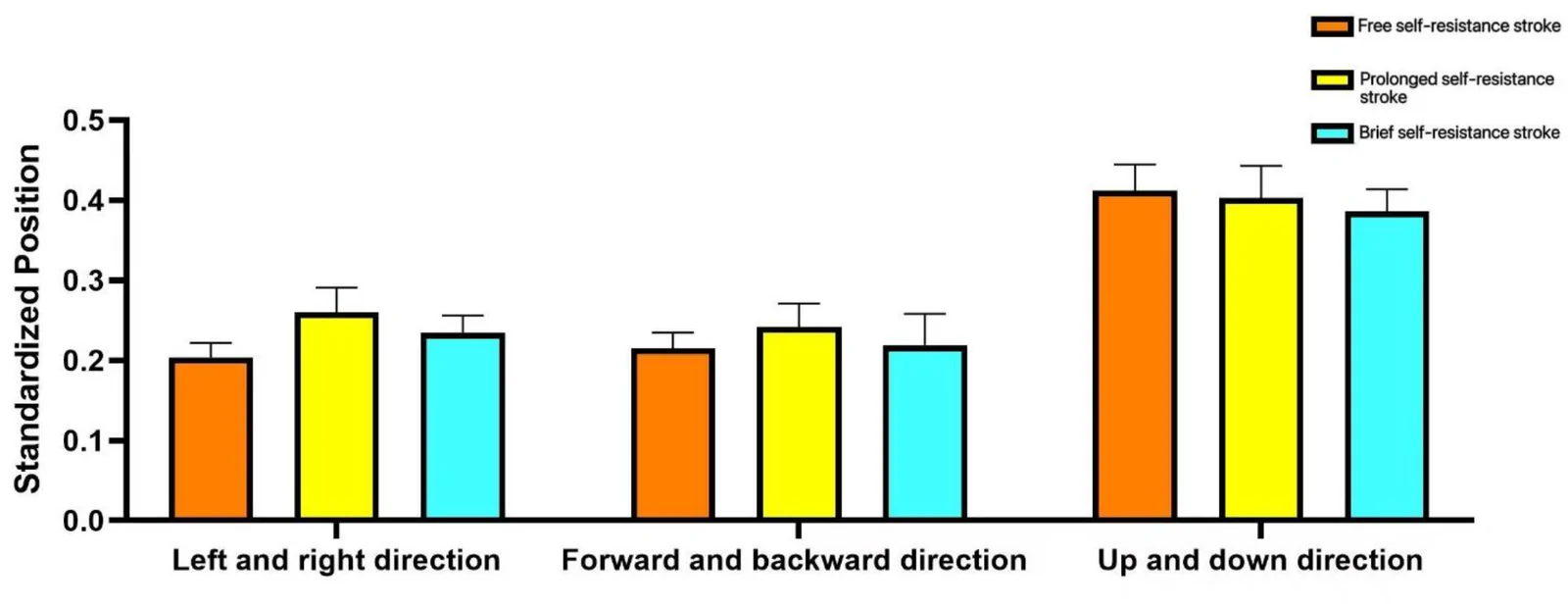
Comparison of the standardized position of the point of contact relative to the toe of the supporting foot for the three stroke types.

#### 3.1.3 Comparison of postural characteristics

Repeated-measures ANOVA showed no statistically significant main effects of strokes on relative center of gravity height (*F* = 0.90, *P* = 0.83, ηp^2^ = 0.03), rear-leg knee angle (*F* = 0.67, *P* = 0.90, *ηp*^2^ = 0.02), supporting-leg knee angle (*F* = 0.95, *P* = 0.76, *ηp*^2^ = 0.03), racket-arm elbow angle (*F* = 0.61, *P* = 0.95, *ηp*^2^ = 0.02), or racket-arm shoulder angle (*F=*0.88, *P* = 0.85, *ηp*^2^ = 0.03). See **Figures 3**, **4**.

**Figure 3:**
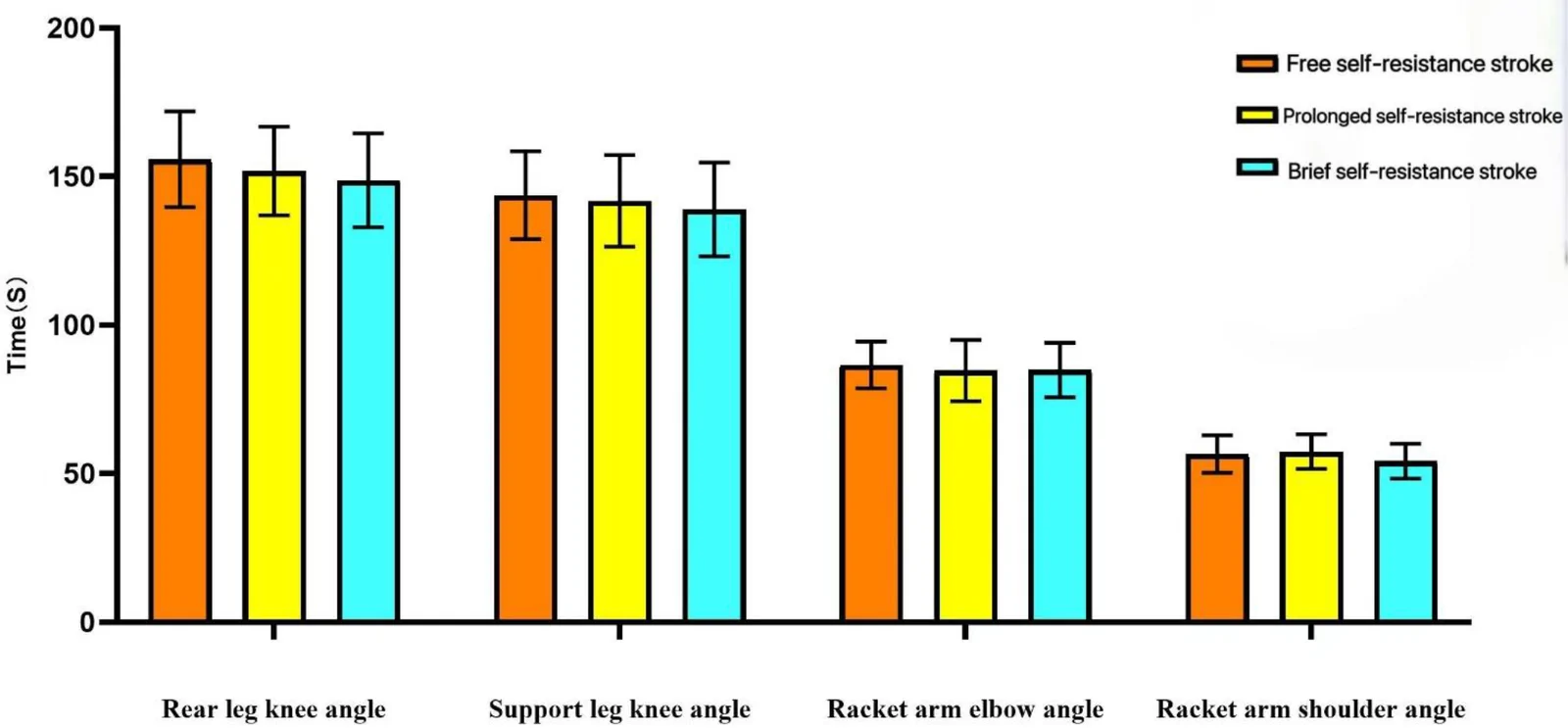
Comparison of postural indicators for strokes phases across three stroke styles (Angles in degrees).

**Figure 4:**
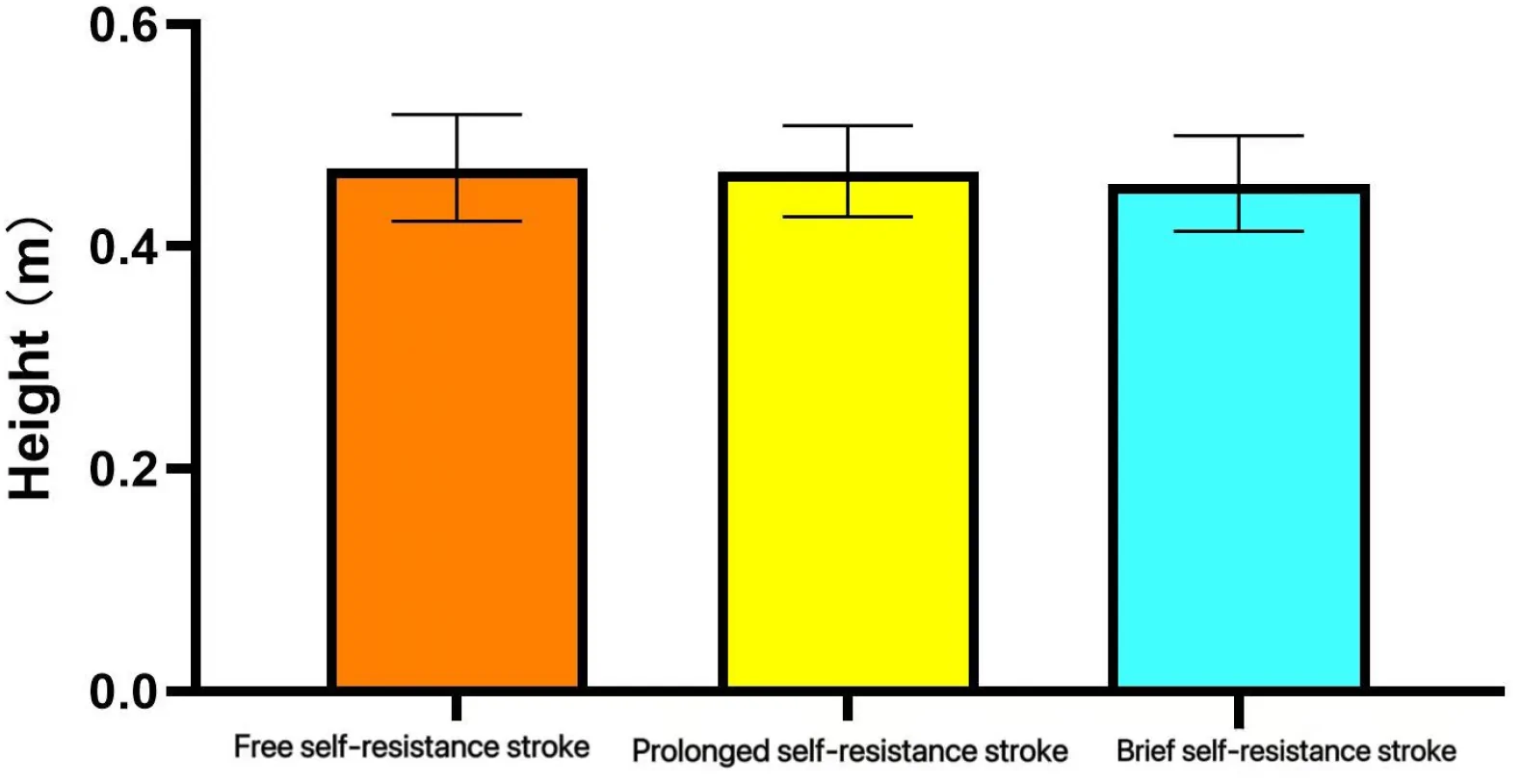
A comparison of the relative height of the center of gravity during three different stroke techniques.

#### 3.1.4 Comparison of ball speed and racket head speed

Statistically significant main effects of strokes were observed for ball speed (*F* = 64.624, *P* < 0.001, *ηp*^2^ = 0.690) and racket head speed (*F* = 3.97, *P* = 0.05, *ηp*^2^ = 0.12). Holm–Bonferroni-adjusted comparisons showed that the brief self-resistance stroke produced a higher ball speed than both the free self-resistance stroke (mean difference = 2.72, 95% CI:2.44 ~ 3.00, *P* < 0.001) and prolonged self-resistance stroke (mean difference = 3.66, 95% CI:3.38 ~ 3.94, *P* < 0.001). Racket head speed was higher in the prolonged and brief self-resistance strokes compared with the free self-resistance stroke (mean difference =1.21, 95% CI:0.88 ~ 1.54, *P* < 0.05; mean difference =1.3, 95% CI:0.94 ~ 1.66, *P* < 0.05). See **Figure 5**.

**Figure 5:**
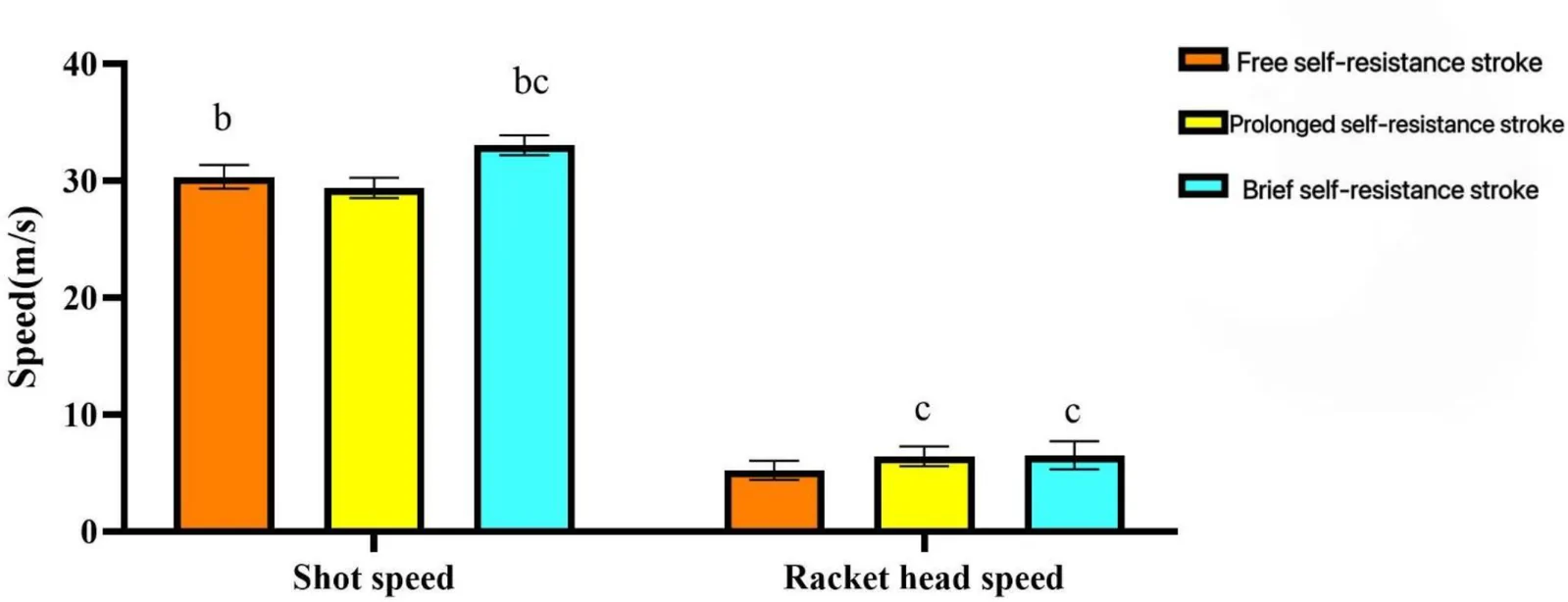
Comparison of ball speed after leaving the racket and racket head speed at the moment of impact for three different stroke types.

**Figure 6:**
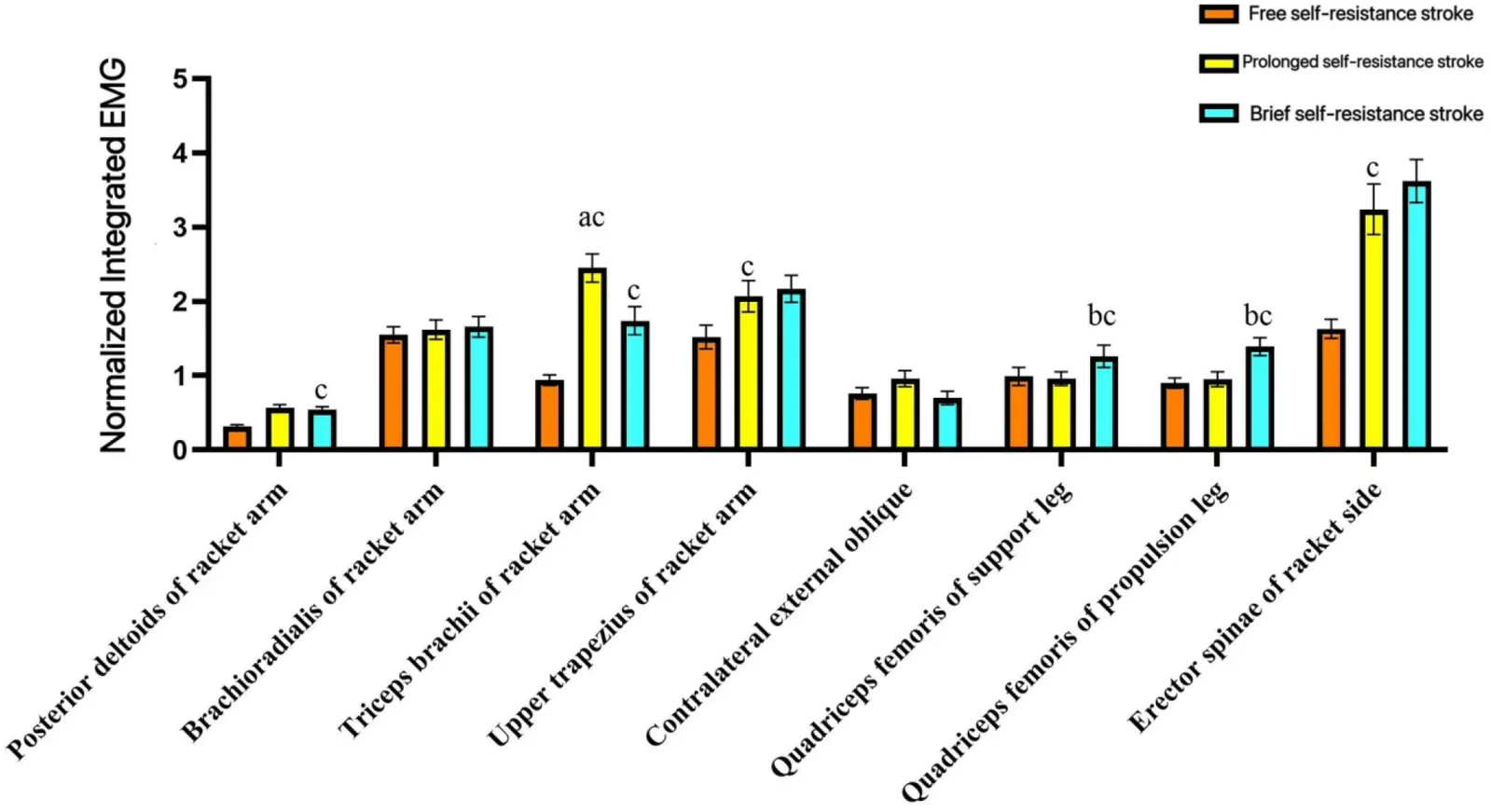
Comparison of integrated electromyographic signals of prime movers for three stroke types.

### 3.2 Comparative analysis of surface electromyography in different muscle activation patterns

#### 3.2.1 Comparison of activation sequences

The results showed that, except for the sequence of muscle activation during the counter-stroke, where the triceps brachii of the racket arm fired earlier than the brachioradialis of the racket arm (P< 0.05), there were no changes in the order of activation for other muscles; muscle activation for the two strokes was grouped into three clusters, while that for the free self-resistance stroke was grouped into two clusters. See **Table 2**.

**Table 2:** Comparison of the timing and sequence of motor unit firing during three different stroke techniques (timing units: s).

Muscle	Free self-resistance stroke			Prolonged self-resistance stroke			Brief self-resistance stroke		
	Activation time	Phase sequence	Activation sequence	Activation time	Phase sequence	Activation sequence	Activation time	Phase sequence	Activation sequence
Posterior deltoids of racket arm	0.76 ± 0.09	8	2	0.75 ± 0.08	8	3	0.60 ± 0.07	8	3
Brachioradialis of racket arm	0.08 ± 0.015	1	1	0.23 ± 0.035a	2	2	0.19 ± 0.03 a	2	2
Triceps brachii of racket arm	0.10± 0.014	2	1	0.05 ± 0.01	1	1	0.03 ± 0.008	1	1
Upper trapezius of racket arm	0.73 ± 0.09	7	2	0.65 ± 0.08	7	3	0.52 ± 0.06	7	3
Contralateral external oblique	0.71 ± 0.08	6	2	0.59 ± 0.07	6	3	0.49 ± 0.06	6	3
Quadriceps femoris of support leg	0.53 ± 0.06ab	3	2	0.45 ± 0.05 ab	3	3	0.38 ± 0.04 ab	3	3
Quadriceps femoris of propulsion leg	0.64 ± 0.07	4	2	0.49 ± 0.06	4	3	0.42 ± 0.05	4	3
Erector spinae of racket side	0.68 ± 0.08	5	2	0.54 ± 0.06	5	3	0.45 ± 0.05	5	3

Activation clusters were determined based on statistical comparisons of muscle activation onset times. Muscles showing no statistically significant differences in activation onset timing were assigned to the same cluster. The activation sequence represents the chronological order of these activation clusters during the stroke phase and is used for descriptive purposes only. Superscript “a” indicates a significant difference in activation onset timing compared with the triceps brachii of the racket-holding arm, and “b” indicates a significant difference compared with the brachioradialis of the racket-holding arm.

#### 3.2.2 Comparison of integrated electromyography values

Statistically significant differences among strokes were observed for several muscles. The iEMG of the posterior deltoid (*F* = 4.00, *P* = 0.04, *ηp*^2^ = 0.12), triceps brachii (*F* = 8.62, *P* < 0.001, *ηp*^2^ = 0.23), upper trapezius (*F* = 4.35, *P* = 0.02, *ηp*^2^ = 0.13), supporting-leg quadriceps (*F* = 4.01, *P* = 0.04, *ηp*^2^ = 0.12), propulsion-leg quadriceps (*F* = 4.74, *P* = 0.04, *ηp*^2^ = 0.14), and erector spinae (*F* = 14.24, *P* < 0.001, *ηp*^2^ = 0.33) differed significantly among strokes. Holm–Bonferroni-adjusted pairwise comparisons indicated that the iEMG values of the posterior deltoid, triceps brachii, upper trapezius, and erector spinae were significantly higher in both resistance-based strokes (prolonged and brief self-resistance strokes) compared with the free self-resistance stroke. Specifically, the prolonged and brief self-resistance strokes showed greater iEMG values for the posterior deltoid (mean differences = 0.25, 95% CI: 0.18~0.32, *P*<0.05; mean differences = 0.22, 95% CI: 0.15–0.29, *P*<0.05), triceps brachii (mean differences = 1.51, 95% CI: 1.28~1.74, *P*<0.05; mean differences = 0.80, 95% CI: 0.57~1.03, *P*<0.05), upper trapezius (mean differences = 0.55, 95% CI: 0.40~0.70, P<0.05; mean differences = 0.65, 95% CI: 0.50~0.80, *P*<0.05), and erector spinae (mean differences = 1.61, 95% CI: 1.35~1.87, *P*<0.05; mean differences = 1.99, 95% CI: 1.70~2.28, *P*<0.05) than the free self-resistance stroke. Furthermore, the brief self-resistance stroke exhibited significantly higher iEMG values than both the free and prolonged self-resistance strokes for the supporting-leg quadriceps (mean differences = 0.27, 95% CI: 0.15~0.39, P<0.05; mean differences = 0.30, 95% CI: 0.18~0.42, *P*<0.05) and propulsion-leg quadriceps (mean differences = 0.49, 95% CI: 0.38~0.60, *P*<0.05; mean differences = 0.44, 95% CI: 0.32~0.56, *P*<0.05). These findings indicate that resistance-based stroke strategies were associated with greater cumulative neuromuscular activity in specific prime movers, with the brief self-resistance stroke showing the greatest increase in lower-limb muscle iEMG.

## 4 Discussion

This study compared the kinematic and surface electromyographic characteristics of three types of one-handed tennis backhand strokes performed under different self-resistance strokes: a free self-resistance stroke with minimal involvement of the non-racket-holding hand, a prolonged self-resistance stroke involving sustained resistance before release, and a brief self-resistance stroke involving brief and rapid resistance before release. The findings indicate that although the three strokes showed similar movement structures, differences were observed in movement rhythm, stroke performance, and neuromuscular activation patterns.

### 4.1 Influence of different self-resistance strategies on movement rhythm and hitting performance during stroke execution

This study found that while there were no differences in the point of contact or body posture among the three stroke types, the movement rhythm varied. Specifically, the duration of the rotational phase in the brief self-resistance stroke was shorter than that in the prolonged stroke and the free self-resistance stroke. These results suggest that the spatial characteristics of the stroke are relatively fixed, while the temporal characteristics are obviously regulated by muscle activation patterns.

This study observed that brief self-resistance stroke achieved the fastest rotational rhythm. This may be because the brief self-resistance stroke pattern approximates a highly compressed preload-release process. Following the brief resistance phase, the muscles controlling the racket may have experienced a transient pre-activation state, which could contribute to faster subsequent rotational movement. This phenomenon bears conceptual similarities to the classic stretch-shortening cycle (SSC) model (Bosco et al., 1982; Komi, 2000; Trimble et al., 2000) described in previous work (Fukutani et al., 2020), which may partly account for its compressed rotational phase duration, thereby achieving faster rotational strokes. However, the experimental design of this study does not yet allow for direct verification of the SSC mechanism, and this requires confirmation in future research using more direct electromyographic or biomechanical measurement methods. In contrast, the free self-resistance stroke contains no preloading stimulus. Without preliminary bilateral counteraction, muscle activation magnitude was lower during rotation, which coincided with prolonged rotational phase duration. Based on the prolonged self-resistance stroke, the experiment revealed that the speed of the head strike was not proportional to the ball speed. This may be due to a certain degree of stress relaxation in the elastic components of the muscle-tendon complex resulting from sustained resistance, thereby preventing the full utilisation of the previously accumulated elastic potential energy during the subsequent concentric contraction.

From the perspective of racket-head and ball velocity after impact, coordinated whole-body momentum transmission determines stroke performance (Kibler, 1995). Rapid torso rotation shortens movement latency and enables sequential activation of lower-limb, trunk and upper-limb muscles, forming a continuous kinetic chain that yields higher post-impact ball speed (Martin et al., 2014; Kwon, 2026). Therefore, the brief self-resistance stroke demonstrated altered temporal movement patterns and superior ball velocity in this acute hitting task. Whether this strategy can enhance training adaptations or competitive performance requires further longitudinal investigation.

### 4.2 Influence of different self-resistance strokes on stroke performance and ball-striking outcomes

In this study, the racket head speed for both types of self-resistance strokes was greater than that for free self-resistance stroke; however, the ball speed for the brief self-resistance stroke was higher than that for the prolonged self-resistance stroke, with the latter’s ball speed even falling below that of the free self-resistance stroke. This seemingly contradictory result—where the prolonged self-resistance stroke, despite having a higher racket head speed, produced the lowest ball speed—precisely reveals the decisive influence of the preload duration on energy transfer efficiency in muscle function.

Firstly, the higher racket head speed observed in self-resistance strokes may be attributed to the fact that, compared to unopposed strokes, the muscles are already under a certain load prior to impact. The brain sends stronger neural drive signals in advance, recruiting more and larger motor units, thereby inducing higher phase-normalized cumulative iEMG amplitudes of the main propulsive muscles (Bobbert et al., 1997; Aagaard et al., 2002; Duchateau et al., 2006). However, these neural and mechanical processes were not directly measured in the present study, and this interpretation should therefore be considered as a possible explanation rather than a demonstrated mechanism. At the same time, the preload stretches the muscle spindles, which may induce a stretch reflex, further enhancing the excitability of α-motor neurons (Calancie and Bawa, 1985) Consequently, both resistance-based stroke techniques can accumulate larger pre-impact iEMG amplitude of agonist muscles, which ultimately raises racket head speed. However, prolonged self-resistance stroke results in significant energy loss at the moment of impact. The possible reason is that from a musculoskeletal mechanics perspective, muscles are viscoelastic bodies; sustained manual holding leads to the gradual dissipation of elastic potential energy and a reduction in the stiffness of the tendon-muscle complex (Proske and Morgan, 2001; Flanagan and Comyns, 2008). In contrast, the preload duration for transient resistance is extremely brief; this may activate the stretch reflex and store elastic potential energy (Rahimi et al., 2006), thereby preventing energy loss and achieving a higher ball speed. However, whether elastic energy storage or utilization contributed to the observed increase in ball speed requires further verification using direct kinetic and neuromuscular measurements. Although the energy conversion efficiency of the unresisted strokes is within the normal range, the lack of preload results in lower pre-impact muscle activation level and reduced cumulative iEMG magnitude, leading to the slowest racket head speed and sub-optimal ball speed. This interpretation should also be treated cautiously, as the present study lacked direct kinetic measurements to quantify mechanical energy transmission across body segments.

### 4.3 Effects of different self-resistance strokes on muscle activation sequence and integrated electromyographic signals

The surface electromyography results from this study show that the sequence of muscle activation during free self-resistance stroke can be divided into two clusters, while the two types of resistance hitting present three clusters. One possible interpretation is that the varying degrees of free-hand involvement prior to the stroke may lead the central nervous system to adopt differentiated motor unit recruitment strategies during the subsequent stroke. Previous studies (Komi et al., 2000; Linnamo et al., 2003) have indicated that under preloaded strokes, neural drive patterns may exhibit more complex temporal characteristics, which is conceptually consistent with our observation of additional activation clusters in the self-resistance strokes. Furthermore, it is worth noting that for both types of self-resistance strokes, the triceps brachii of the racket arm was activated earlier than the brachioradialis, whereas the opposite was observed in free self-resistance stroke. This phenomenon may tentatively suggest that the presence of preload prompts the elbow extensor group to activate earlier, potentially to counteract the release of external resistance and thereby establish a more stable force couple for the subsequent stroke. It should be emphasized, however, that these interpretations are speculative and derived from existing physiological models; our experimental design does not directly verify changes in neural drive strategy. Future studies incorporating refined EMG timing analysis or neuromuscular stimulation techniques are warranted to substantiate these inferences.

In terms of integrated electromyography (EMG), there were no significant differences across the three stroke types in the iEMG values of the brachioradialis on the racket-holding side and the external oblique on the non-racket-holding side during the ball-enveloping and trunk rotation phases. This suggests that the cumulative neuromuscular activity of these muscles during these phases was relatively consistent across strokes. However, the iEMG values of the prime movers involved in resistance generation, including the posterior deltoid, triceps brachii, upper trapezius, and erector spinae, were higher in the two self-resistance strokes than in the free self-resistance condition. These findings indicate greater cumulative neuromuscular demand during resistance-based strokes. However, because iEMG is influenced by both EMG amplitude and phase duration, these differences should not be interpreted as direct evidence of greater muscle activation intensity. Finally, the stroke motions for all three techniques are based on an open kinetic chain comprising the ground, lower limbs, trunk, upper limbs, and racket. The driving force is generated by the body, transmitted through the lower limbs to create a push against the ground, and the resulting reaction force is then transferred via the lower limbs to the racket head to strike the ball. The lower limbs serve as the pivotal hub for force transmission throughout the entire kinetic chain (Girard et al., 2007; Johnson et al., 2021; Kwon, 2026), and the work performed by the relevant prime movers (the quadriceps muscles of both legs, as tested in the experiment) is essential for the effective execution of the technique. The experimental results showed that the iEMG values of the quadriceps muscles in both legs were highest during brief self-resistance strokes. This finding suggests greater cumulative neuromuscular activity of the lower-limb muscles during this condition. However, whether this elevated quadriceps iEMG corresponds to optimized sequential muscle activation and energy transmission along the kinetic chain requires further verification via kinetic testing and time-normalized EMG analysis.

This study has several limitations. First, the sample consisted exclusively of right-handed male athletes, which may limit the generalizability of the findings to female players or left-handed individuals. Second, the experimental setting, including the use of a ball machine and standardized target areas, may not fully replicate the variability and unpredictability of match-play strokes. Additionally, although high-speed cameras (200 Hz) provided adequate temporal resolution for stroke analysis, manual identification of movement events and frame-by-frame digitization may introduce small measurement uncertainties. Therefore, the reported kinematic variables should be interpreted considering potential errors associated with event identification and coordinate reconstruction. Third, the analysis focused on immediate stroke performance without examining potential fatigue effects or long-term training adaptations across different self-resistance strokes. Fourth, surface EMG cannot fully capture deep muscle activity or distinguish between synergistic contributions. Moreover, the muscle selection in the present study was limited to eight major prime movers associated with upper-limb control, trunk rotation, and lower-limb force transmission during the one-handed backhand stroke. Muscles that contribute to racket-face control and eccentric stabilization, such as the forearm pronators/supinators and shoulder external rotators, were not included. Therefore, the current findings may not fully reflect the contribution of all muscle groups involved in racket control and deceleration during stroke execution. Future studies should incorporate these specific muscle groups to provide a more comprehensive assessment of neuromuscular control during the one-handed backhand stroke. Furthermore, the present study used phase-based iEMG analysis without time normalization. Therefore, future studies should incorporate mean normalized RMS EMG or equivalent time-window analysis to better isolate changes in muscle activation amplitude, and the current study did not include inverse dynamics or joint moment calculations to further validate the kinetic chain interpretation. Fifth, although the three experimental strokes were designed to represent different self-resistance strategies, the magnitude and duration of resistance applied by the non-racket-holding hand were not objectively quantified. Therefore, these should not be interpreted as directly representing specific contraction modes such as sustained manual holding or stretch-shortening cycle activity. Future studies should incorporate force sensors, instrumented rackets, or synchronized kinetic measurements to objectively characterize the mechanical characteristics of each condition.

## 5 Conclusion

No statistically significant differences were detected in the measured movement structure variables among the three stroke types, although they differed in movement rhythm. The brief self-resistance stroke showed the shortest rotational phase, which corresponds to faster torso rotation and higher ball exit velocity upon impact. Racket head speed exceeds that of a free self-resistance stroke, and ball speed peaks at contact. Free self-resistance stroke activation involves two prime-mover muscle groups, whereas stroke involves three, indicating greater complexity and tighter rhythm. The three strokes demonstrated similar movement structures but differed in movement rhythm, stroke performance, and cumulative neuromuscular activity. Brief self-resistance stroke produced the fastest rotational phase and the highest ball speed under the present experimental strokes, suggesting that this resistance strategy may have the potential to enhance acute stroke execution characteristics. However, whether this strategy can translate into improved competitive performance or long-term training benefits requires further investigation. Resistance-based strokes exhibited greater cumulative neuromuscular activity in specific prime movers, although these findings should be interpreted cautiously because iEMG reflects both EMG amplitude and phase duration. Future studies incorporating time-normalized EMG and kinetic measurements are required to further elucidate the mechanisms underlying these performance differences.

## Data Availability

The raw data supporting the conclusions of this article will be made available by the authors, without undue reservation.
